# Correlation between HER2/neu protein overexpression on Immunohistochemistry and Fluorescent in Situ Hybridization (FISH) in breast carcinoma: Problems in developing countries

**DOI:** 10.12669/pjms.39.6.6704

**Published:** 2023

**Authors:** Zubaria Mukhtar, Amina Faisal, Ghazala Mudassir, Nadira Mamoon

**Affiliations:** 1 Zubaria Mukhtar, FCPS Department of Histopathology, Shifa International Hospital, Islamabad, Pakistan; 2 Amina Faisal, Resident Histopathology Department of Histopathology, Shifa International Hospital, Islamabad, Pakistan; 3 Ghazala Mudassir, M.Phil. Department of Histopathology, Shifa International Hospital, Islamabad, Pakistan; 4 Nadira Mamoon, FCPS, FRCPath Department of Histopathology, Shifa International Hospital, Islamabad, Pakistan

**Keywords:** HER2/neu, Fluorescence in situ hybridization (FISH), Immunohistochemistry (IHC)

## Abstract

**Objective:**

To correlate the results of HER2/neu protein overexpression on immunohistochemistry (IHC) and gene amplification on fluorescence in situ hybridization (FISH) and to document the problems faced in performing FISH procedure.

**Methods:**

This was an observational retrospective study covering five years from January 1^st^, 2015 - December 31^st^, 2019 at Histopathology Department of Shifa International Hospital (SIH), Islamabad. All cases of breast cancer that underwent florescence in situ hybridization (FISH) were retrieved. Correlation between HER2/neu overexpression on IHC and its amplification on FISH was analyzed. Problems in application of FISH were recorded.

**Results:**

Out of 451 cases submitted for HER2/neu testing by FISH, 68 cases (15%) were rejected. Gene amplification was seen in 139 (36.29%) cases. Total cases with HER2/neu IHC score of 2+ were 330 cases and out of which gene amplification was seen in 98 cases (29.69%) whereas 93.1% (41/44) 3+ IHC cases were amplified. Poor fixation, inadequate amount of tumor with crushing artefacts and dye application to the biopsy fragments were causes of sample rejection.

**Conclusions:**

Her2/neu amplification was seen in most Her2/neu 3+ cases and approximately one-third of Her2neu 2+ cases. Proper fixation, adequate biopsy material with standardized processing is required to yield useful results on FISH.

## INTRODUCTION

Breast carcinoma comprises 22.9% of the malignant tumors world-wide and 16% of all the cancers occurring in females. Global cancer statistics have depicted a high incidence and mortality from breast carcinoma in Europe and America.^1^ A meta-analysis of ten studies on cancer incidence in Pakistan revealed the prevalence of breast cancer in females ranged from 20 to 50% with an overall prevalence of 31%.^2^ The third highest incidence of breast cancer for any Asian population is reported in Karachi.^3^ Over-expression of HER2/neu protein or amplification of HER2/neu gene has been detected in 30% to 40% of breast carcinomas and is associated with poor prognosis and decreased survival.^4^,^5^

Antimonoclonal HER2/neu antibody (Herceptin) is found to prolong survival^.^ This therapy is effective only in those cases where HER2/neu status is accurately detected. HER2/neu status can accurately be detected by several methods like polymerase chain reaction (PCR), immunohistochemistry (IHC), fluorescence in situ hybridization (FISH), and chromogenic insitu hybridization (CISH).^6^ However the two methods mainly in use are IHC for protein over expression and FISH for gene amplification. Our study aimed to evaluate the results of HER2/neu testing by FISH in our hospital and correlate it with IHC scores. This study also aimed to highlight the problems which interfere in the interpretation of IHC and FISH results and reasons for specimen rejection.

## METHODS

We conducted a retrospective study at histopathology department of Shifa International Hospital (SIH), Islamabad after approval of Institutional review board and ethical committee vide their letter no IRB#290-780-219 dated November 26, 2019. A total of 451 diagnosed cases of breast cancer underwent florescence in situ hybridization (FISH) in a five-year period (1^st^ January 2015-31^st^ December 2019). Formalin fixed and paraffin embedded blocks of both core and excision biopsies were analyzed for correlation between HER2/neu overexpression on IHC and its amplification on FISH and these were assessed according to the American Society of Clinical Oncology and the College of American Pathologists (ASCO/CAP) guidelines, 2018.

### Immunohistochemical (IHC) processing and interpretation:

IHC for HER2/neu protein was performed on 2-3-micron thick paraffin embedded tissue sections placed on poly-L-lysine coated slides. Following deparaffinization and blocking of endogenous peroxidase, HER2/neu was done by applying c-erbB2 oncoprotein as primary antibody (Roche) on Ventana platform automated IHC stainer. Diaminobenzidine (DAB) was added as chromogen. Each slide was reviewed by a pathologist and immunostaining was read in semi quantitative manner according to ASCO/CAP recommended guidelines. IHC scores 0 and 1+ were designated as negative expression and 3+ were designated as positive expression. Score 2+, the grey zone area, was taken as equivocal and these slides were reviewed by two different pathologists who if reaffirmed the score of 2+ was further subjected to FISH testing.

### Fluorescence in situ hybridization (FISH) analysis and interpretation:

FISH analysis was done using a dual HER2/CEP17 probe (Path Vysion HER2 DNA Probe kit, Abbott Molecular) combining HER2 gene probe (locus specific identifier, LSI) with a centromeric enumeration probe for chromosome 17 (CEP17). Sections of target tissue were cut 4-5microns thick on coated slides from paraffin embedded blocks and baked overnight at 56°C. The slides were then deparaffinized in xylene, dehydrated in 100% ethanol and air dried. Later on, these slides were placed in pretreatment solution (sodium thiocyanite) and then in protease solution already placed in water bath at 37Ċ for 25 minutes. It was followed by dehydration using 80%, 96% and 100% ethanol in sequential steps for one minute each.

After this, target section was marked, probe was applied and slide was sealed with sealant. It was then placed in hybridizer and program set and run. After that post- hybridization washes were given with post- hybridization wash buffer at room temperature for five minutes. Cover slip was removed and slide placed again in post hybridization wash buffer already placed in water bath at 70 Ċ for five minutes. Slides were recovered from water bath, washed with three changes of distilled water. Dehydration was done in 70%, 86% and 96% ethanol. They were dried completely in dark, DAPI was applied and cover slip was gently placed. Control was run with each batch.

Slides were screened under fluorescent microscope (Olympus BX 51) using appropriate filters (DAPI, FITC, TRITC dual and triple band pass filters). Signals were counted in at least 20 nuclei and scored for green and red signals. Red signals represent HER2 gene copies and green signals represent CEP17 gene copies. The mean number of CEP17 and HER2 signals was recorded and results were expressed as a ratio of red to green signals as per ASCO/CAP recommended guidelines.

### Inclusion Criteria:

All cases of breast carcinoma were included in this study.

### Exclusion Criteria:

None of the breast carcinoma cases was excluded as we wanted to document the factors leading to rejection of samples.

## RESULTS

**Fig.1:**
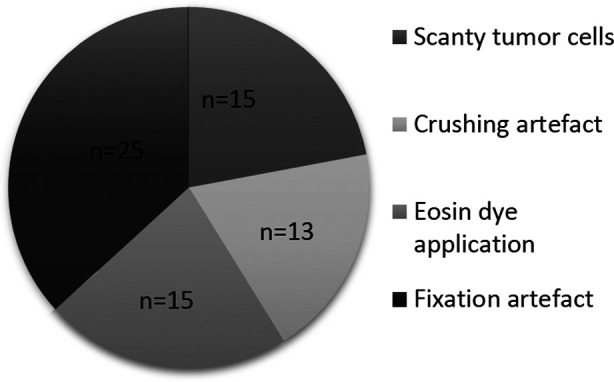
Reasons for specimen rejection.

A total of 451 cases of breast carcinoma were submitted for florescence in situ hybridization (FISH) within the study period. IHC results were available for all except two cases. Out of 451 cases, acceptable results for FISH were obtained in 383 cases (85%) while 68 cases (15%) were rejected due to various reasons (Fig.1).

Out of total 68 rejected cases, 25 cases (40%) were received as blocks processed from outside labs while 14 cases (20%) were of samples processed in SIH. The exact source of the block could not be traced in 27 cases. Mean age of the patients was 51.4 ± 12.4 years. Two hundred cases were of core biopsies and 183 were excision specimens. Sample source was SIH (in-house) in 230 cases (60.0%) and 153 cases (40.0%) were received from outside collection points. Out of these in 75 cases (20%) were received as pre-formed blocks.

**Fig.2:**
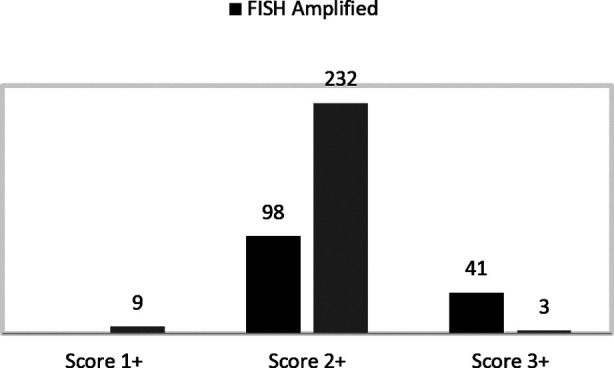
Correlation between HER2 IHC scoring and gene amplification on 383 cases.

Out of a total of 383 cases, HER2/neu IHC was performed at SIH in 339 cases (88.5%) and in 44 cases (11.5%) it was performed at some outside laboratory. Gene amplification was seen in 139 (36.5%) cases and result was negative in 244 (63.5%) cases. Total cases with HER2/neu IHC score of 2+ were 330 and out of these, gene amplification was seen in 98 cases (29.7%), while 232 cases (70.3%) were FISH negative. However, 93.1% (41/44) 3+ IHC cases were amplified. Correlation between HER2 IHC scoring and gene amplification is shown (Fig.2).

## DISCUSSION

HER2/neu testing in breast cancer is a sensitive subject. It is a requirement for applying the relatively new molecular classification to breast cancer.^7^,^8^ HER2/neu gene (also known as ERBB-2) is located on the long arm of chromosome 17 (17q12-21.32) which encodes for oncoprotein p185. This acts as receptor tyrosine kinase and is linked with multiple signal transduction pathways. This oncoprotein has seen to be overexpressed in different human cancers other than breast also. These include ovarian, gastric, pancreatic, prostatic, colorectal and lung cancer.

Approximately 25-30% of the breast cancers have shown overexpression of HER2neu gene. This overexpression of the gene is a poor prognostic factor and a significant predictor for response to humanized recombinant monoclonal antibody to HER2/neu; trastuzumab (Herceptin).^4^,^5^ This forms the basis of accurately classifying patients for HER2 /neu targeted therapy. Herceptin (trastuzumab) is globally approved for use as an adjuvant therapeutic agent for breast cancer patients with HER2/neu over expression. Therefore, accurate evaluation of HER2/neu status is of prime importance in patient selection for HER2 targeted therapy.

We studied HER2 gene amplification using FISH in Pakistani breast cancer patients. ASCO and CAP guidelines have mentioned the attainment of a concordance level of at least 95% between IHC and FISH results. However, in practical terms, concordance between IHC and FISH results may vary from 50-100%.^9^ The results of our study showed a concordance level of 93% on FISH testing for positive cases with HER2/neu 3+ on IHC (41/44 cases). All of our cases (9) scored as 1+ were negative for gene amplification on FISH.

In our study three cases (0.07%) which scored 3+ on IHC for HER2 did not show any gene amplification on FISH which was an unusual observation. This may be attributed to artefactual high sensitivity of immunohistochemical assays or gene amplification below the level of detection of FISH essay. Similar observations have been reported in other studies as well.^10^-^12^ Goud et al^13^ reported seven cases with the score of 3+ by IHC which were negative for gene amplification on FISH and suggested that all cases showing a 3+ score by IHC should go for FISH to rule out polysomy of chromosome 17 which could be falsely interpreted as HER2/neu overexpression by IHC analysis.

Majority (330) of our FISH cases were reported as 2+ on immunohistochemistry which were examined by at least two pathologists. Cases referred for FISH from outside the hospital had IHC already performed outside. Performing FISH on all IHC2+ cases is essential as these patients may benefit from targeted immunotherapy. However, the reporting of cases in the 2+ category requires stringent perception and experience. Majority of these cases were processed in our hospital setting. Out of these cases only 98 cases (29.7%) were detected positive for HER2/neu gene amplification and a significant proportion, 232/330 (70.3%) cases were negative. These figures correlate well with previous studies.^14^,^15^ A study done by Horimoto^16^ had 11% of IHC 2+ cases with FISH amplification. Another study in India reported 68.8% of cases reported as IHC 2+ of which 44% were FISH amplified and 53.6% were gene non amplified.^17^

Breast cancer is diverse in its morphological appearance and this tumor heterogeneity poses difficulty not only in its accurate diagnosis and definitive typing but also in assessment of immunohistochemistry results of HER2/neu. This may be a significant factor affecting interpretation of HER2/neu status on IHC as well as in FISH and is frequently detected among HER2/neu equivocal cases (IHC score 2+).^18^

The results of our study also highlighted the importance of internal quality control along with external quality control programs (CAP & ISO) with which we are affiliated. Pre-analytical factors can pose difficulty not only in procedure execution but also in result interpretation. As FISH is a very sensitive technique, any deviation from standard protocols for specimen fixation and processing may hamper the results. ASCO/CAP and NCCN guidelines have also addressed this fact. It is justified to reject samples for HER2/neu IHC and FISH testing if certain pre-analytical conditions are not met. These conditions include prolonged cold ischemia time, inappropriate fixative type and short fixation time.^18^,^19^

**Fig.3:**
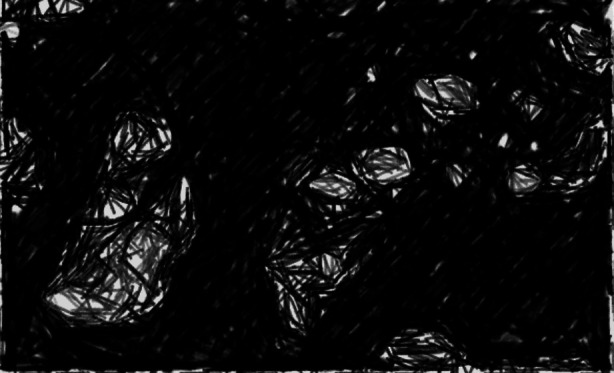
Interference with red signals interpretation due to eosin dye application.

A significant proportion of cases submitted for FISH testing in our study were rejected due to various reasons. Out of these, suboptimal/delayed fixation was found to be the most important factor which interferes with the results. We found it very challenging to maintain the standard guidelines for specimen processing as we receive specimens from different locations across the country with no control over cold ischemia time, quality of fixative and duration of fixation. Similarly, another practice of applying eosin or any colored dye to make small cores or fragments visible for sample processing also interferes with red signals of FISH and leads to non-readable results. (Fig.3).

Core biopsies present another challenge as the tumor tissue left may be too scanty or crushed. We should discourage such practices and highlight the importance of proper fixation and processing to all concerned including surgeons, radiologists and pathologists. It is important to emphasize and make surgeons and oncologists aware of these limitations in application of FISH testing.

### Limitations:

A significant limitation of our study was the different sources of the specimens with lack of information as regards fixation times, duration and processing methods of blocks received etc. this information could help to further narrow down causes of sample rejection**.**

## CONCLUSION

HER2/neu gene amplification was seen in 29.7% and 93.1% cases scored as 2+ and 3+ on IHC respectively in this study. These patients with gene amplification can benefit from HER2/neu targeted therapy. All measures need to be taken to establish the reliability of tests undertaken to ascertain the HER2/neu status in breast carcinoma patients. Reliable results of FISH require optimal fixation, adequate amount of tumor tissue without crushing artefacts and no prior dye application to the biopsy fragments.

### Authors Contribution

**ZM:** conceived, designed and did statistical analysis & editing of manuscript, is responsible for integrity of research.

**AF, GM:** did data collection and manuscript writing.

**NM:** did review and final approval of manuscript.
